# Supplementary material for: Fertility protection during chemotherapy treatment by boosting the NAD(P)+ metabolome
Source: EMBO Mol Med. 2024 Aug 21;16(10):17. doi: 10.1038/s44321-024-00119-w (PMC11473878; doi:10.1038/s44321-024-00119-w)
Supplement: Supplementary file 1 — Appendix [file 44321_2024_119_MOESM1_ESM.pdf]

APPENDIX

APPENDIX SUPPLEMENTARY FIGURES 2
Appendix Figure S1. 2
Appendix Figure S2 3
ARRIVE GUIDELINES CHECKLIST 4
LFQ ANALYST REPORT 6

APPENDIX SUPPLEMENTARY FIGURES

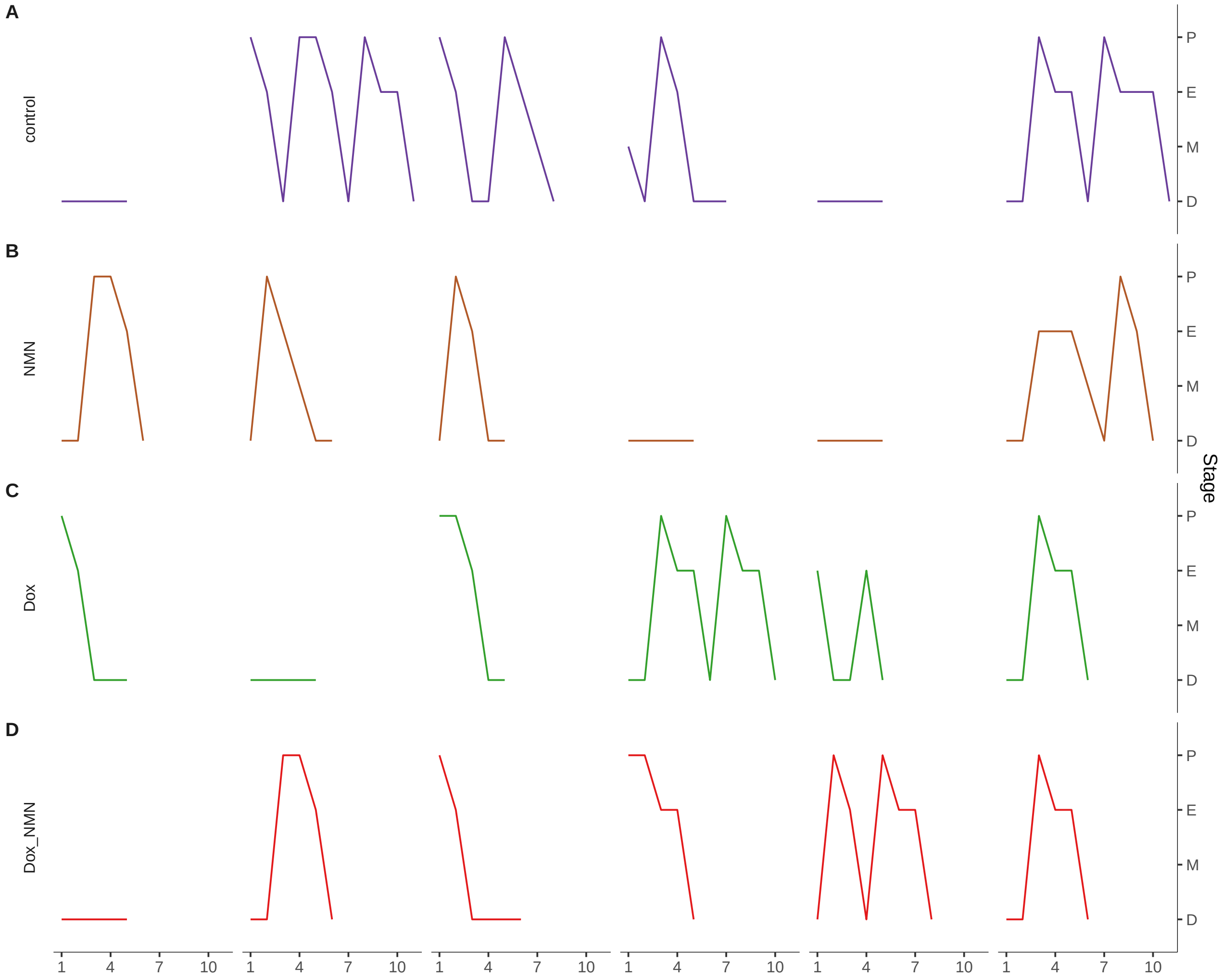

Appendix Figure S1. *Estrous cycling in (A) untreated or treated with (B) NMN, (C) doxorubicin (DOX) treated and (D) DOX with NMN, as described in Fig. 4A. Animals were subject to daily vaginal smearing and cytology to assess estrous cycling, allowing ovarian tissue collection at the diestrus stage, with ovaries subject to stereology and assessment of the ovarian reserve as in Fig. 4. Smears were used to assess whether animals were at proestrus (P), estrus (E), metestrus (M) and diestrus (D) were as described as in Methods.*

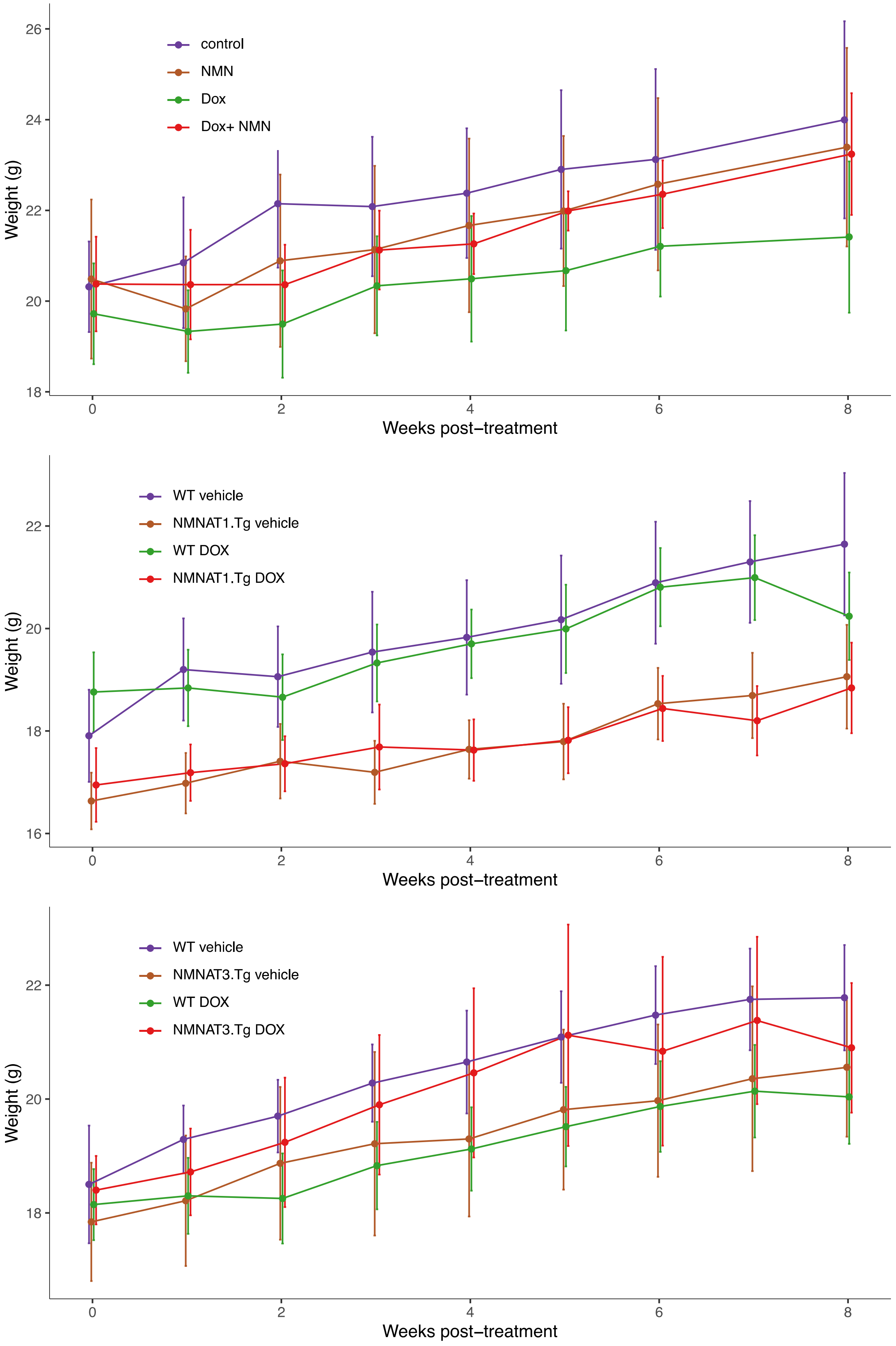

Appendix Figure S2*. Body weights following doxorubicin treatment in A) animals co-treated with NMN, B) NMNAT1 transgenics and C) NMNAT3 transgenics. Error bars are mean ± 95% CI.*

ARRIVE GUIDELINES CHECKLIST

See next page.

NOTE: Please save this file locally before filling in the table, DO NOT work on the file within your internet browser as changes will not be saved. Adobe Acrobat Reader (available free here) is recommended for completion.

The ARRIVE guidelines 2.0: author checklist

The ARRIVE Essential 10

These items are the basic minimum to include in a manuscript. Without this information, readers and reviewers cannot assess the reliability of the findings.

Item		Recommendation	Section/line number, or reason for not reporting
**Study design**	1	For each experiment, provide brief details of study design including:	
		a. The groups being compared, including control groups. If no control group has been used, the rationale should be stated.	Methods (page 22-23) and fig. legends.
		b. The experimental unit (e.g. a single animal, litter, or cage of animals).	Methods, page 33
**Sample size**	2	a. Specify the exact number of experimental units allocated to each group, and the total number in each experiment. Also indicate the total number of animals used.	Fig. legends, results, methods
		b. Explain how the sample size was decided. Provide details of any *a priori* sample size calculation, if done.	Methods, p34
**Inclusion and exclusion criteria**	3	a. Describe any criteria used for including and excluding animals (or experimental units) during the experiment, and data points during the analysis. Specify if these criteria were established *a priori*. If no criteria were set, state this explicitly.	Methods, page 33
		b. For each experimental group, report any animals, experimental units or data points not included in the analysis and explain why. If there were no exclusions, state so.	Fig. 3: two animals excluded, described in legend.
		c. For each analysis, report the exact value of *n* in each experimental group.	Figure legends.
**Randomisation**	4	a. State whether randomisation was used to allocate experimental units to control and treatment groups. If done, provide the method used to generate the randomisation sequence.	Methods, page 33
		b. Describe the strategy used to minimise potential confounders such as the order of treatments and measurements, or animal/cage location. If confounders were not controlled, state this explicitly.	Methods, page 33
**Blinding**	5	Describe who was aware of the group allocation at the different stages of the experiment (during the allocation, the conduct of the experiment, the outcome assessment, and the data analysis).	Methods, p22, 25-26, 28, 29, 30, 33-34.
**Outcome measures**	6	a. Clearly define all outcome measures assessed (e.g. cell death, molecular markers, or behavioural changes).	Figure legends, methods
		b. For hypothesis-testing studies, specify the primary outcome measure, i.e. the outcome measure that was used to determine the sample size.	Figure legends, methods
**Statistical methods**	7	a. Provide details of the statistical methods used for each analysis, including software used.	Figure legends, methods.
		b. Describe any methods used to assess whether the data met the assumptions of the statistical approach, and what was done if the assumptions were not met.	Methods, p34
**Experimental animals**	8	a. Provide species-appropriate details of the animals used, including species, strain and substrain, sex, age or developmental stage, and, if relevant, weight.	Methods (p22), fig legends
		b. Provide further relevant information on the provenance of animals, health/immune status, genetic modification status, genotype, and any previous procedures.	Methods, p22
**Experimental procedures**	9	For each experimental group, including controls, describe the procedures in enough detail to allow others to replicate them, including:	Methods
		a. What was done, how it was done and what was used.	Methods, fig legends
		b. When and how often.	
		c. Where (including detail of any acclimatisation periods).	Methods, p22
		d. Why (provide rationale for procedures).	Methods, results
**Results**	10	For each experiment conducted, including independent replications, report:	
		a. Summary/descriptive statistics for each experimental group, with a measure of variability where applicable (e.g. mean and SD, or median and range).	Figures
		b. If applicable, the effect size with a confidence interval.	Figures - Tukey boxplots

LFQ ANALYST REPORT

Relates to proteomics data in Figure 6 of main manuscript – see next page.

LFQ-Analyst report

03 February, 2023

Method details

The raw data files were analyzed using MaxQuant to obtain protein identifications and their respective label-free quantification values using in-house standard parameters. Of note, the data were normalization based on the assumption that the majority of proteins do not change between the different conditions. Statistical analysis was performed using an in-house generated R script based on the ProteinGroup.txt file. First, contaminant proteins, reverse sequences and proteins identified "only by site" were filtered out. In addition, proteins that have been only identified by a single peptide and proteins not identified/quantified consistantly in same condition have been removed as well. The LFQ data was converted to log2 scale, samples were grouped by conditions and missing values were imputed using the 'Missing not At Random' (MNAR) method, which uses random draws from a left-shifted Gaussian distribution of 1.8 StDev (standard deviation) apart with a width of 0.3. Protein-wise linear models combined with empirical Bayes statistics were used for the differential expression analyses. The *limma* package from R Bioconductor was used to generate a list of differentially expressed proteins for each pair-wise comparison. A cutoff of the *adjusted p-value* of 0.05 (Benjamini-Hochberg method) along with a |log2 fold change| of 1 has been applied to determine significantly regulated proteins in each pairwise comparison.

Quick summary of parameters used:

- Tested pairwise comparisons = ctrl_vs_Dox_NMN, ctrl_vs_Dox, ctrl_vs_NMN, Dox_vs_Dox_NMN, NMN_vs_Dox_NMN, NMN_vs_Dox
- Adjusted *p-value* cutoff <= 0.05
- Log fold change cutoff >= 1

Results

MaxQuant result output contains proteins groups of which *3906* proteins were reproducibly quantified.

154 proteins differ significantly between samples.

Exploratory Analysis (QC Plots)

Principle Component Analysis (PCA) plot

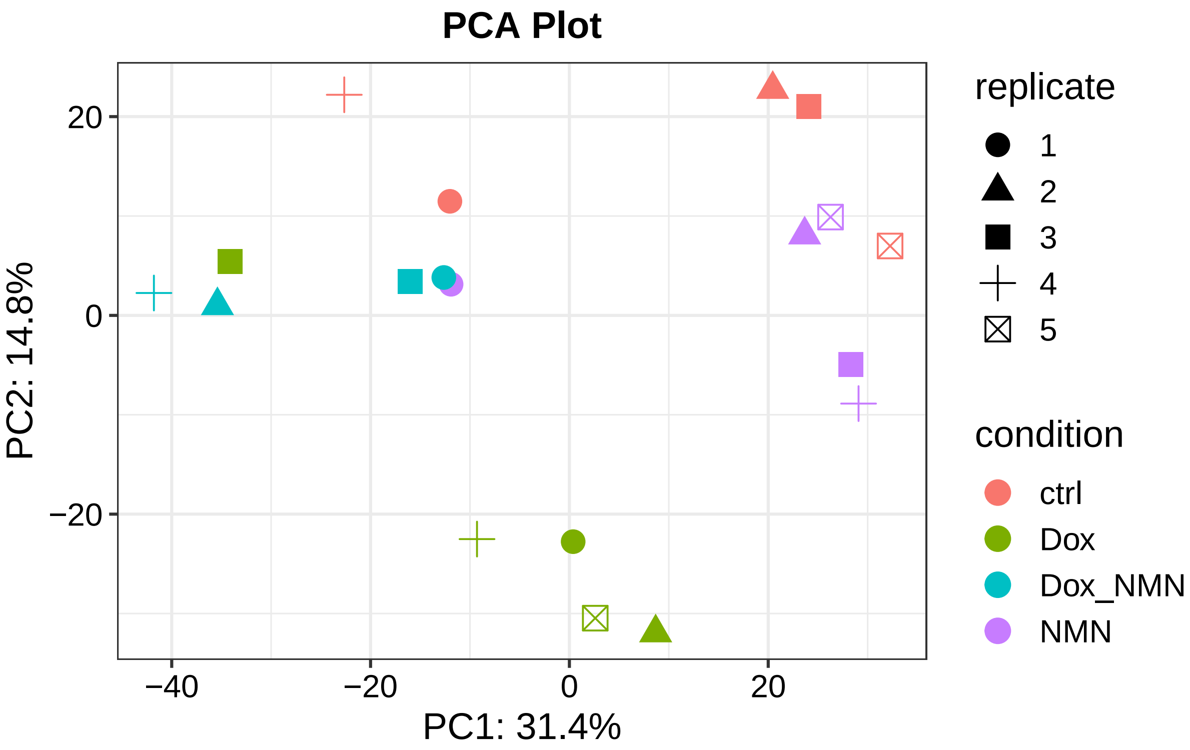

Sample Correlation matrix

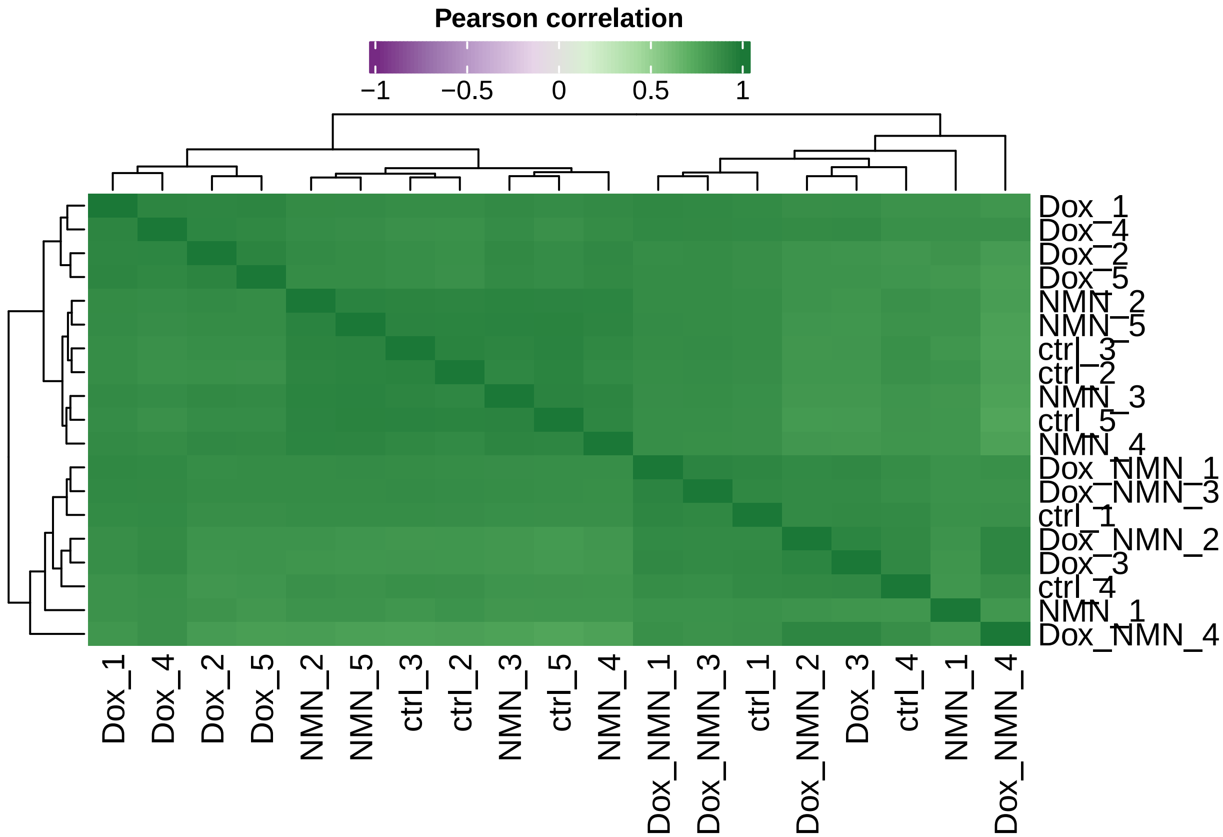

Sample Coefficient of variation (CVs)

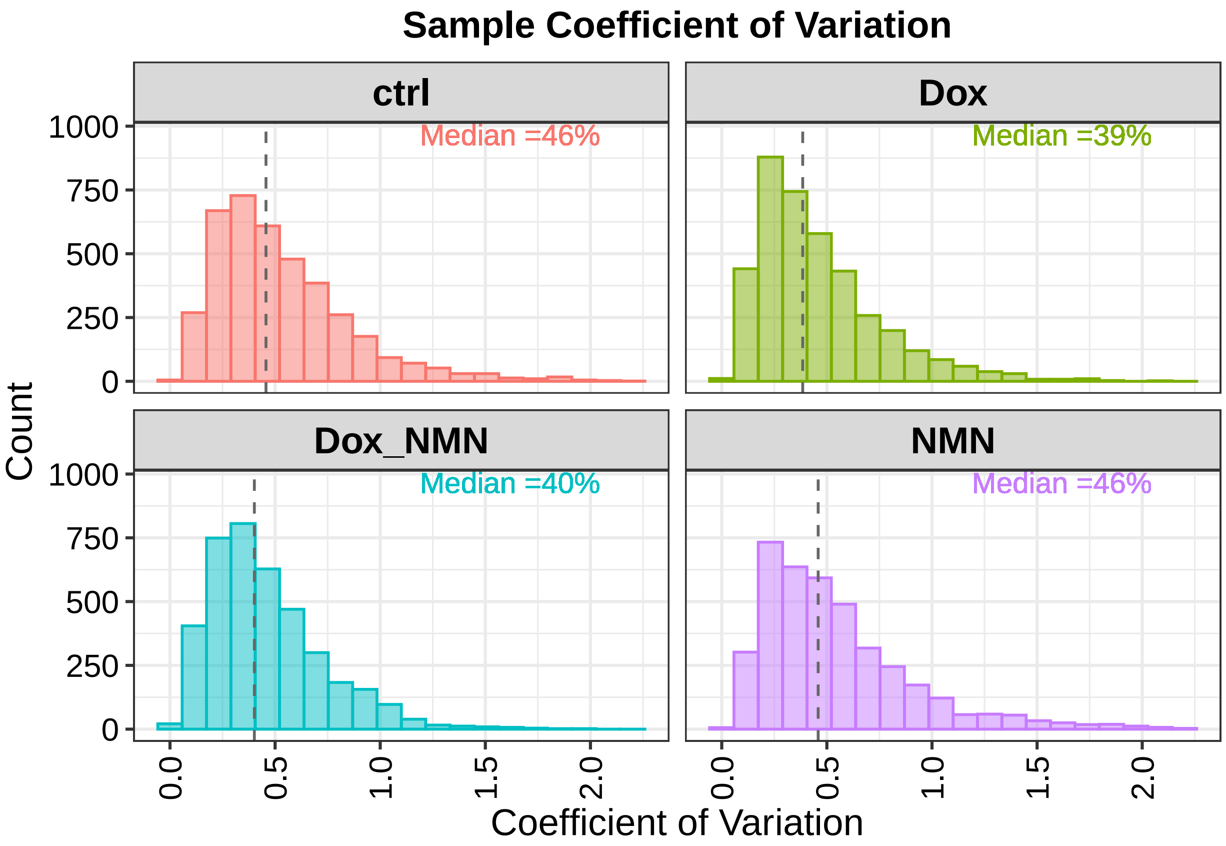

Proteomics Experiment Summary

Protein quantified per sample (after pre-processing).

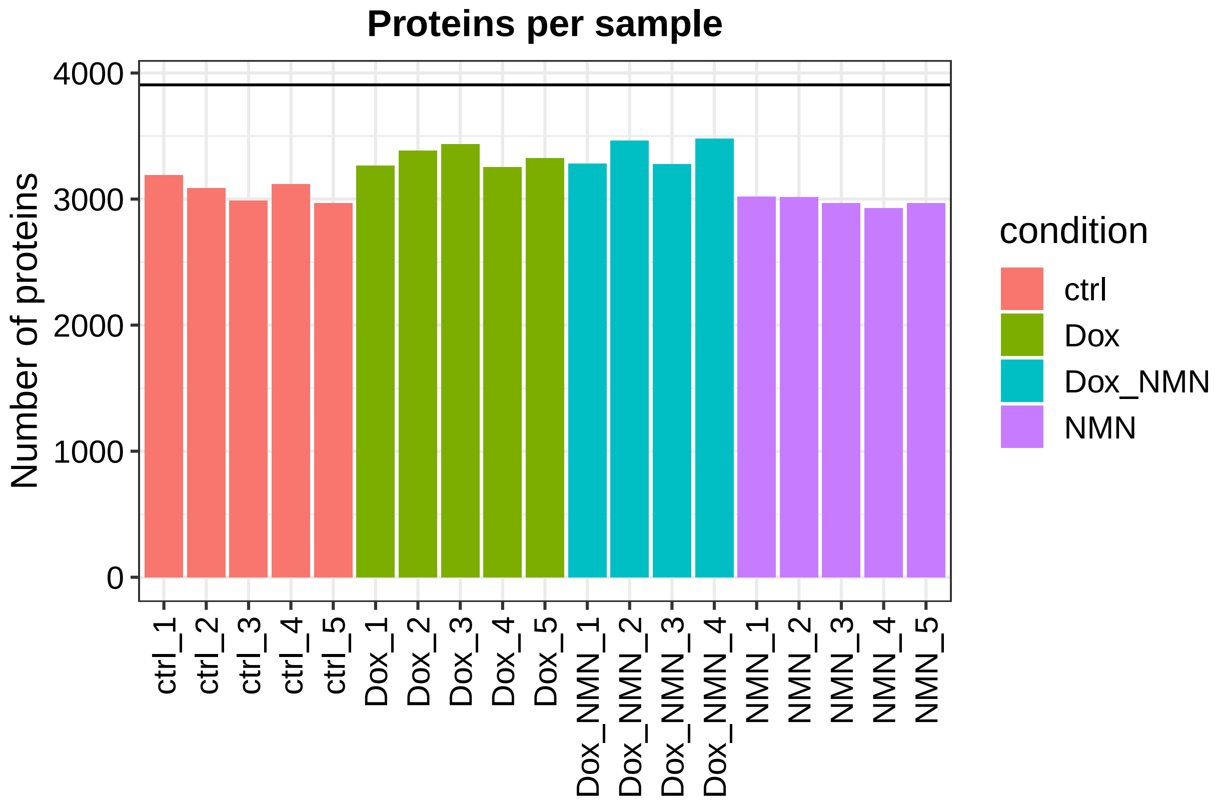

Protein overlap in all samples.

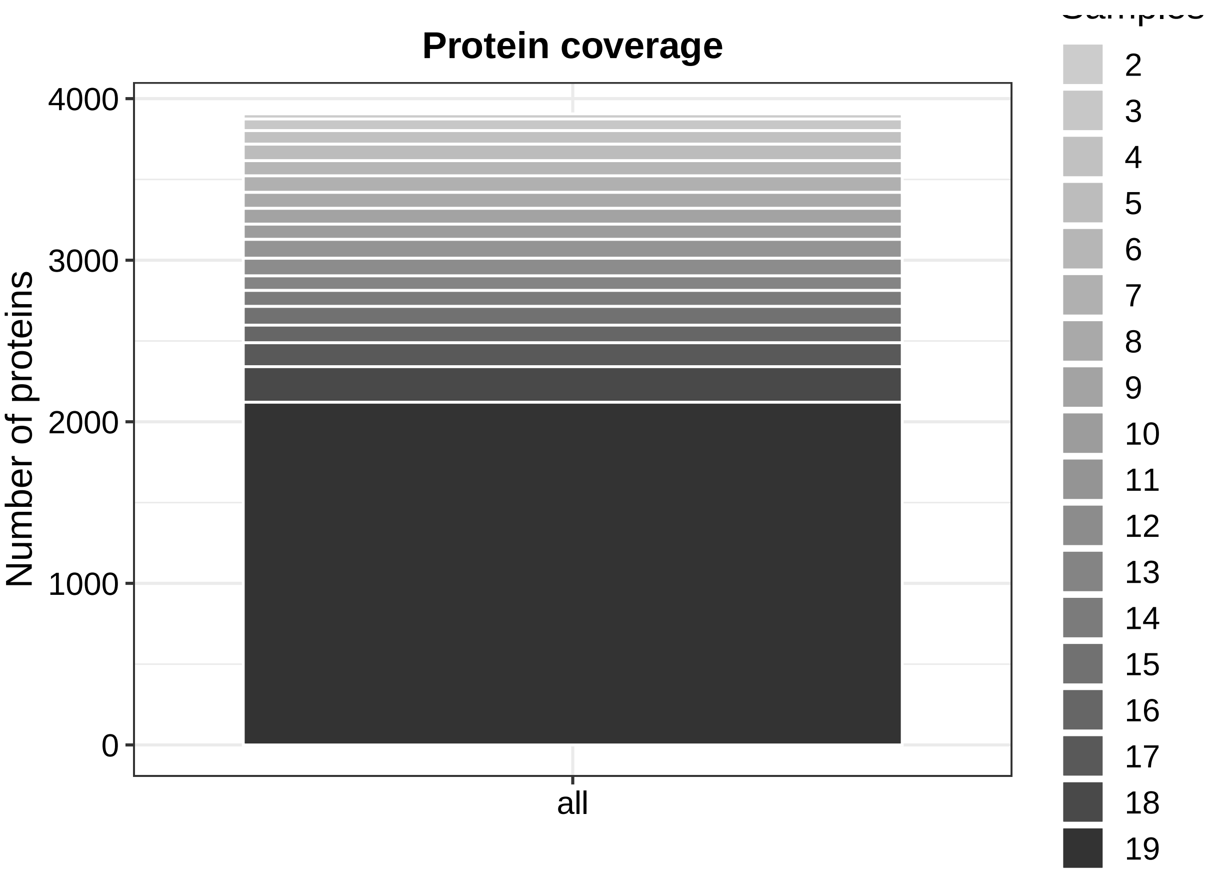

Missing Value handling

Missing value heatmap A heatmap for proteins with missing value in each dataset. Each row represent a protein with missing value in one or more replicate. Each replicate is clustered based on presence of missing values in the sample.

Missing value distribution Protein expression distribution before and after imputation. The plot showing the effect of imputation on protein expression distribution.

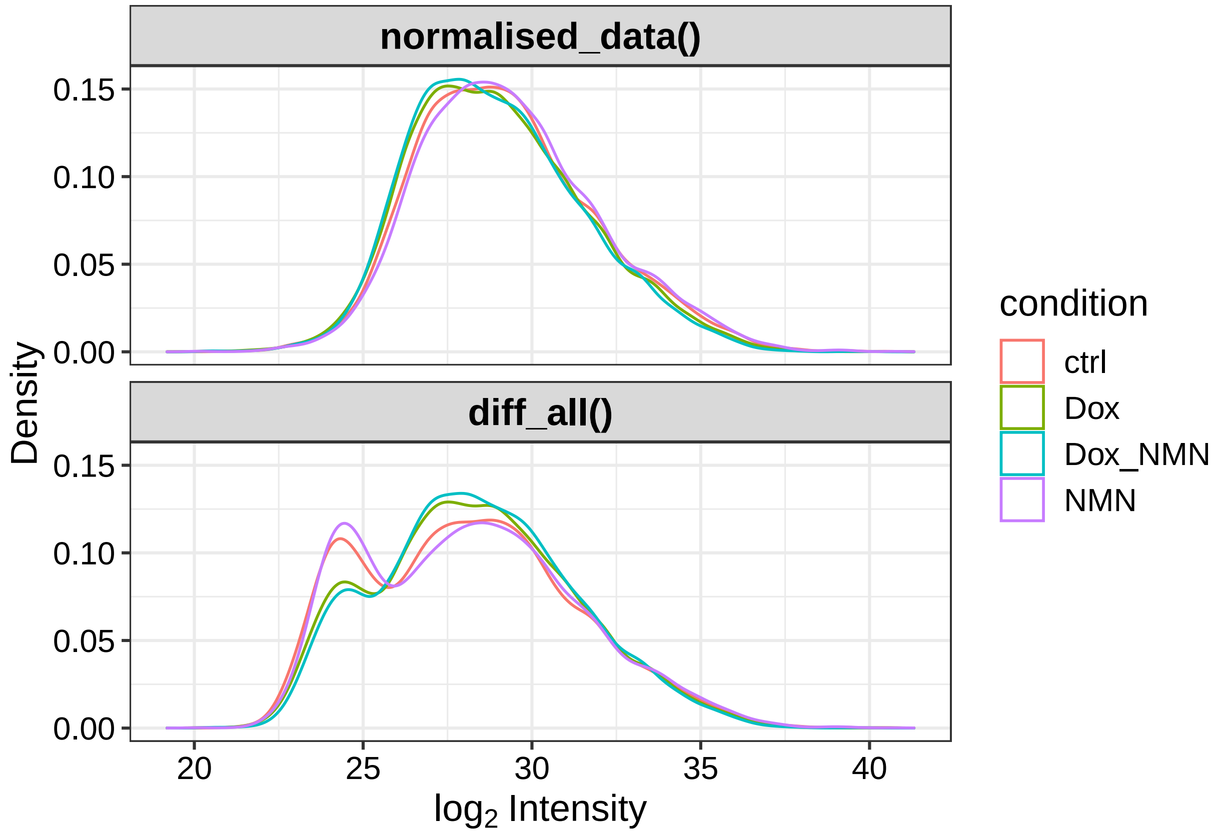

Differential Expression Analysis (Results Plots)

Heatmap A plot representing an overview of expression of all significant (differencially expressed) proteins (rows) in all samples (columns).

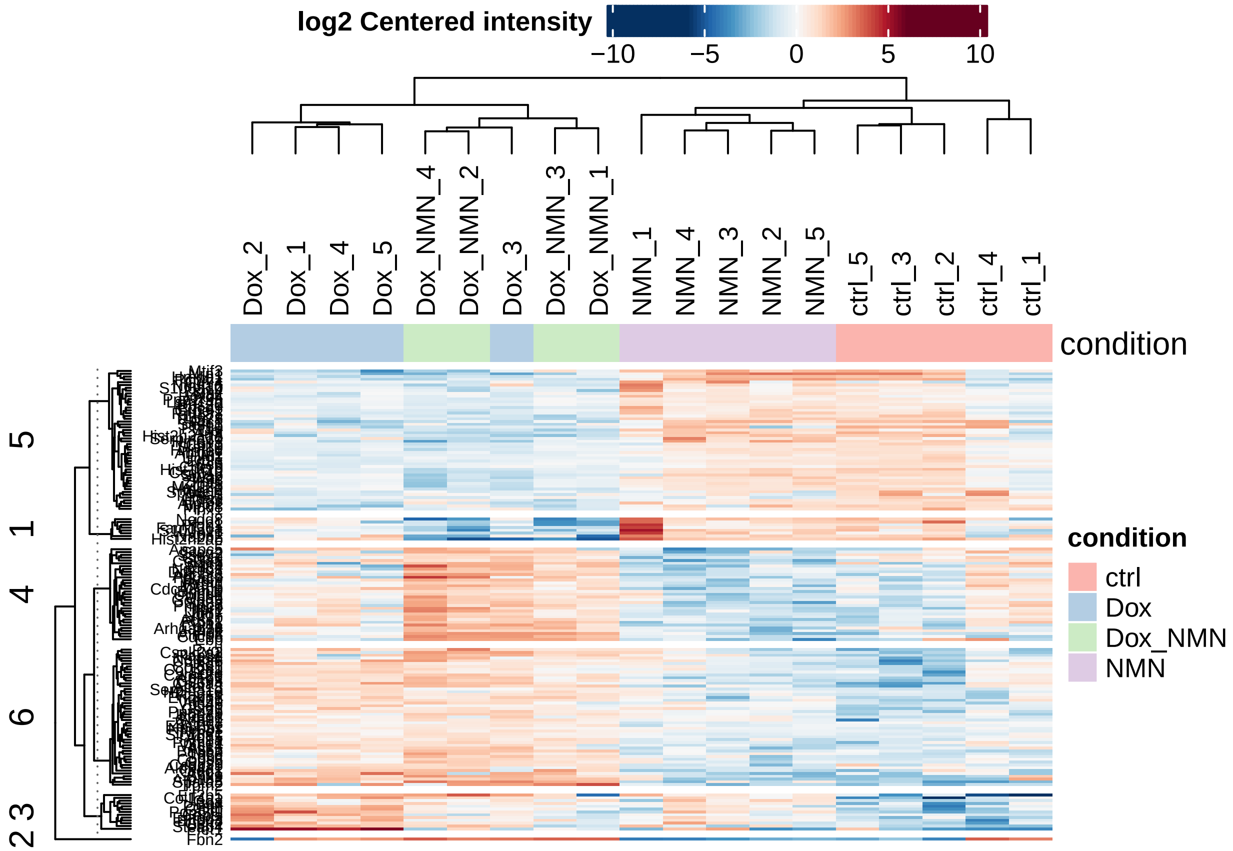

Volcano Plots

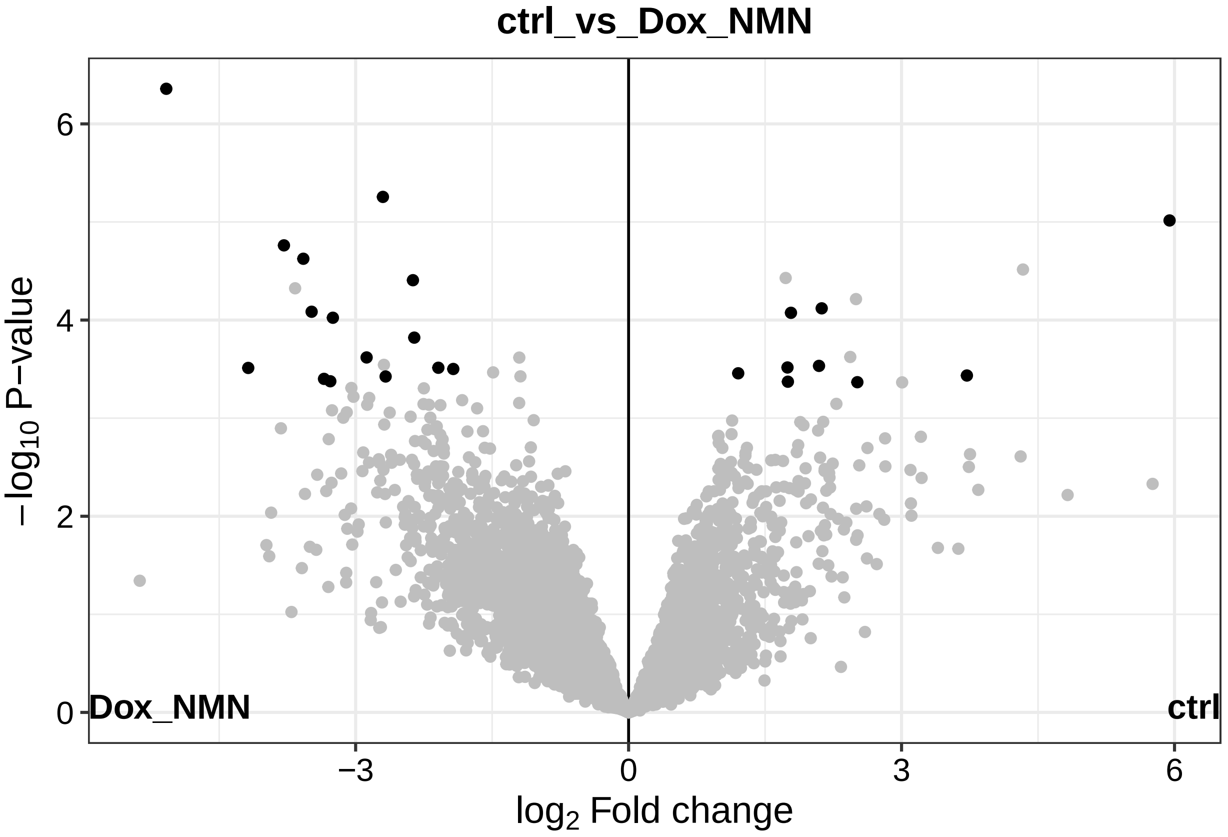

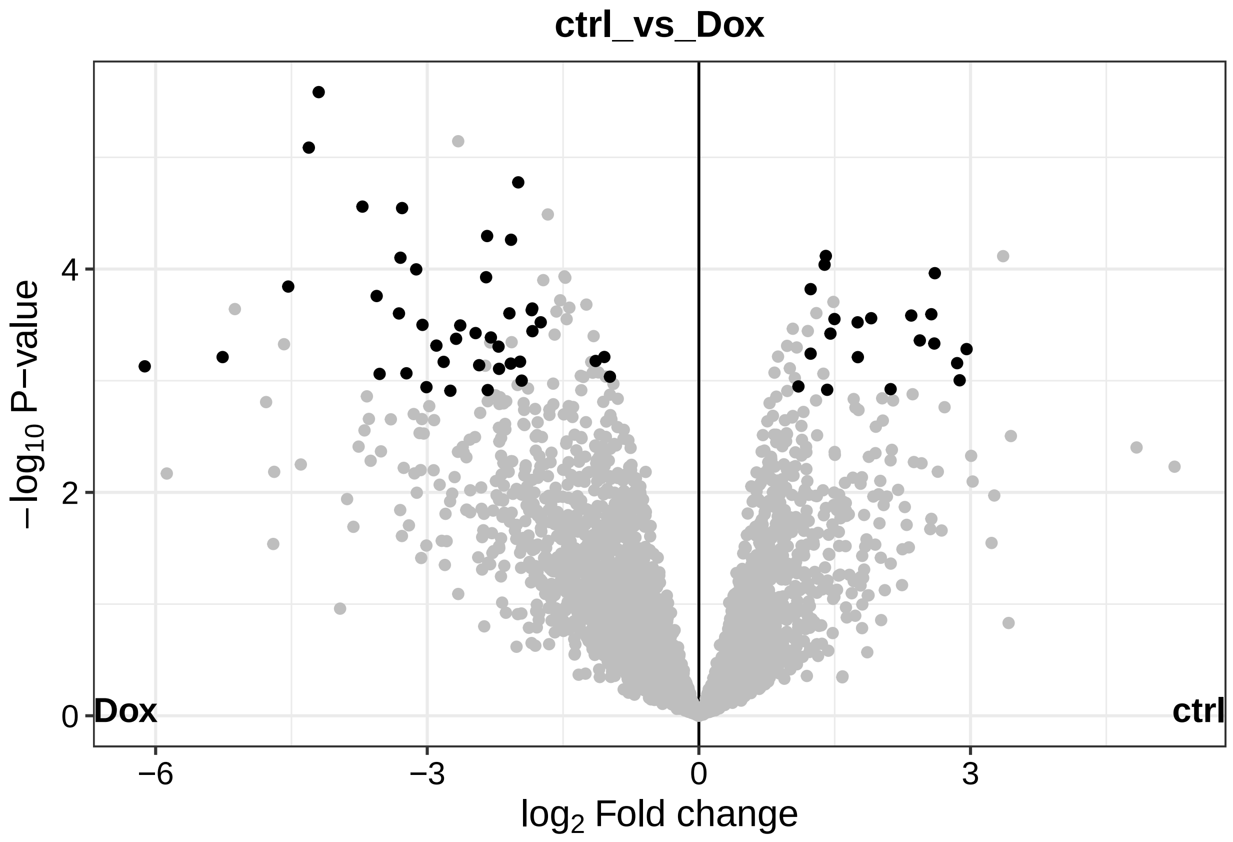
ctrl_vs_Dox
−log10 P−value
log2 Fold change
Dox
ctrl
0
2
4
−6
−3
0
3

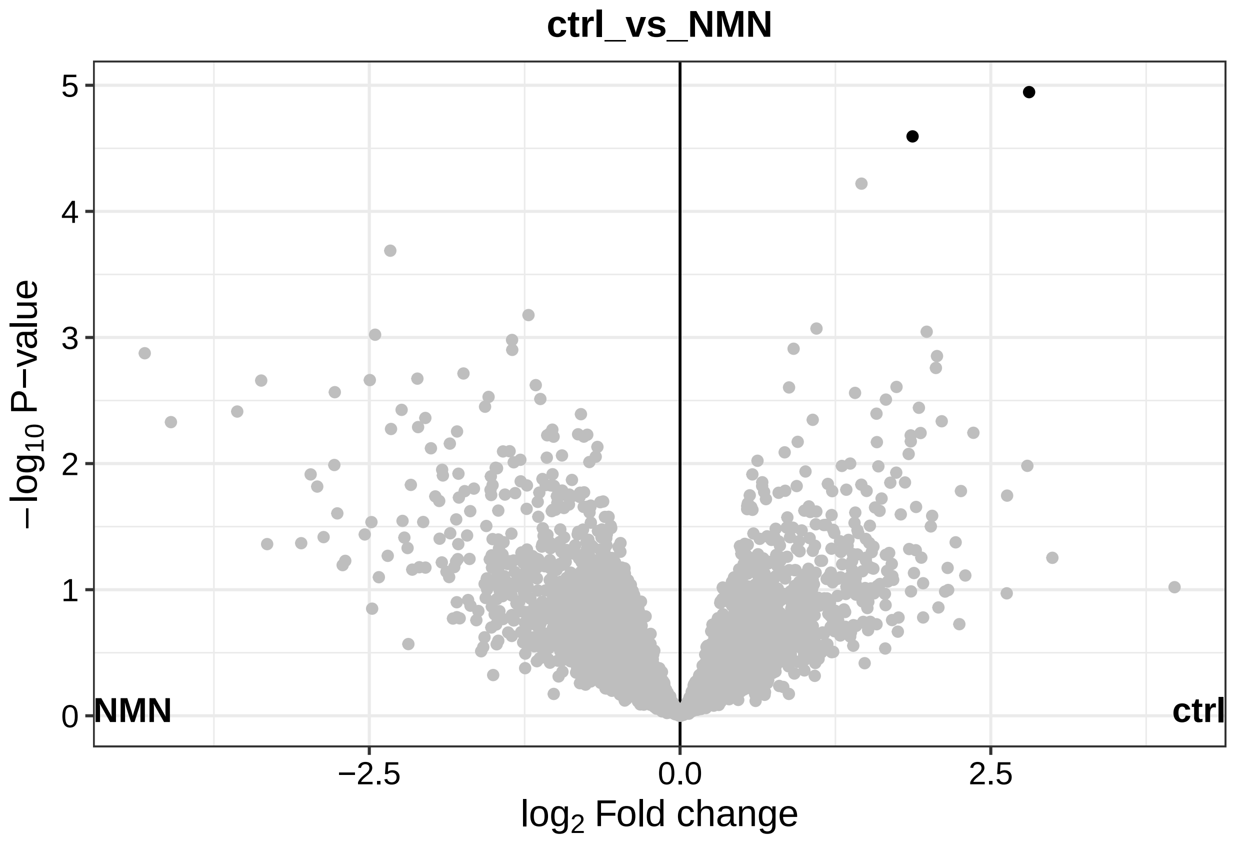
ctrl_vs_NMN
5
4
3
2
1
0
−log10 P−value
NMN
ctrl
−2.5
0.0
2.5
log2 Fold change

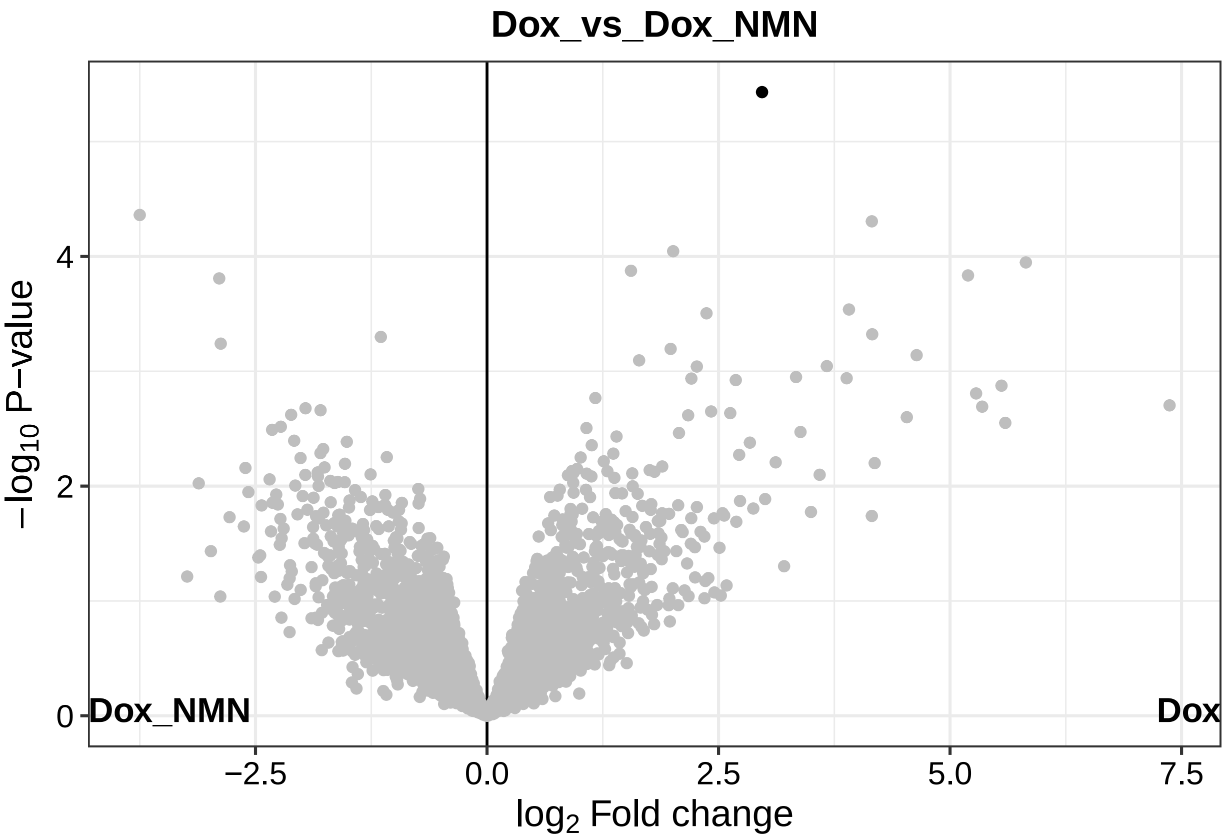
Dox_vs_Dox_NMN
−log10 P−value
log2 Fold change
Dox_NMN
Dox
0
2
4
−2.5
0.0
2.5
5.0
7.5

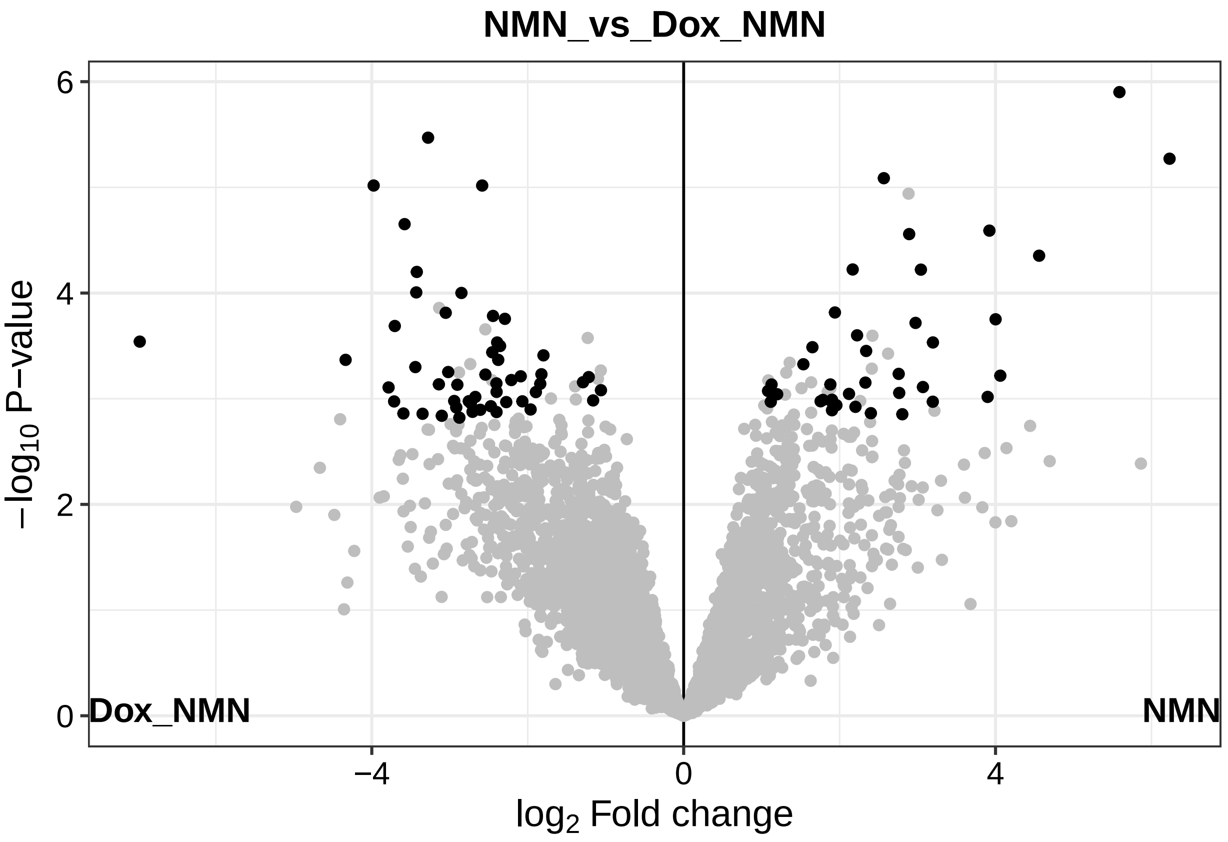

NMN_vs_Dox_NMN
−log10 P−value
6
4
2
0
Dox_NMN
NMN
−4
0
4
log2 Fold change

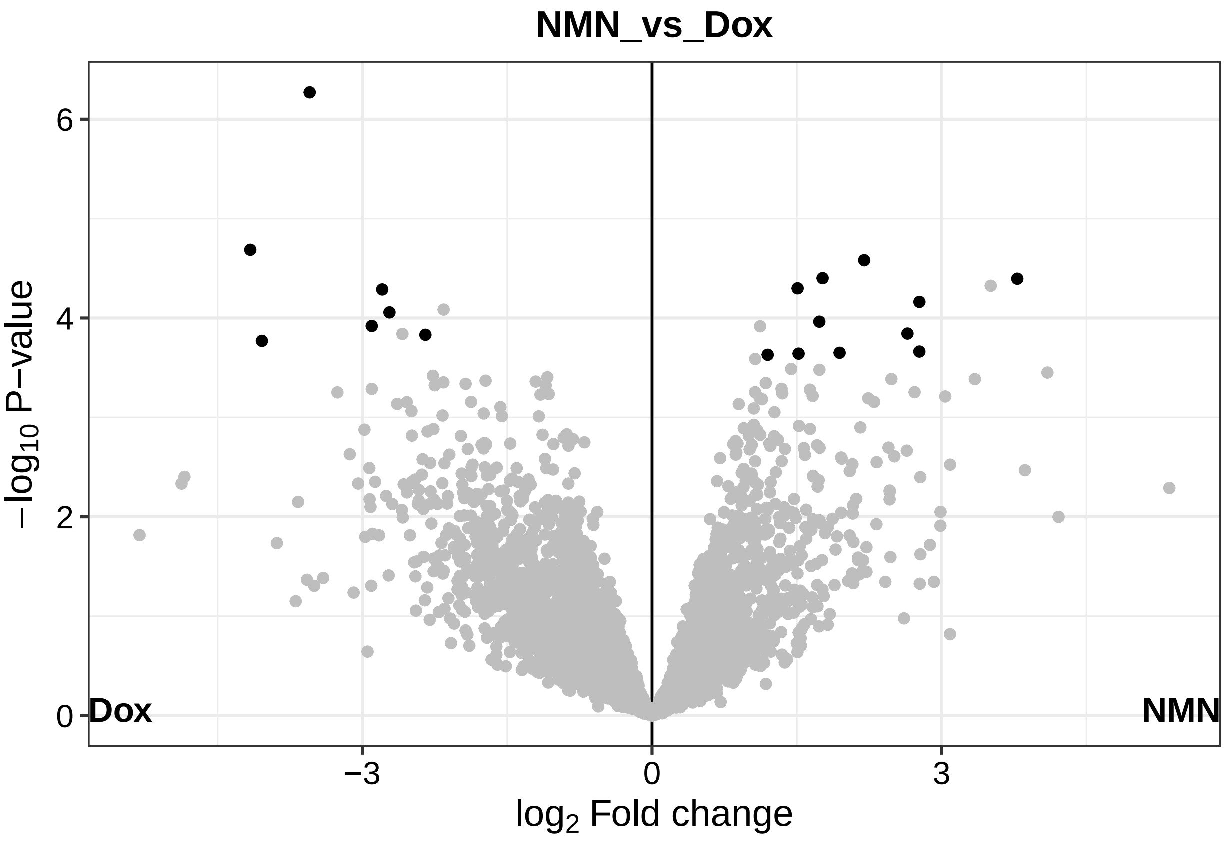
NMN_vs_Dox
−log10 P−value
log2 Fold change
Dox
NMN
6
4
2
0
−3
0
3
