# Supplementary material for: Fertility protection during chemotherapy treatment by boosting the NAD(P)+ metabolome
Source: EMBO Mol Med. 2024 Aug 21;16(10):17. doi: 10.1038/s44321-024-00119-w (PMC11473878; doi:10.1038/s44321-024-00119-w)
Supplement: Supplementary file 3 — Expanded View Figures [file 44321_2024_119_MOESM3_ESM.pdf]

Expanded View Figures

Figure EV1. Kinetics of oocyte maturation.

Germinal vesicle (GV) stage cumulus oocyte complexes (COCs) from animals stimulated with PMSG in main Fig. 1 were collected in media containing IBMX to prevent meiotic maturation, mechanically denuded and moved to IBMX-free media to allow meiotic resumption. The proportion of oocytes undergoing germinal vesicle breakdown (GVBD) and polar body extrusion (PBE) were assessed at the indicated timepoints in oocytes obtained from mice treated with (**A**) doxorubicin (Dox) or (**B**) cisplatin in the presence or absence of NMN, as in main Fig. 1B,C, and in (**C**) NMNAT1 and (**D**) NMNAT3 transgenic or wild-type (WT) littermates treated with Dox, as in main Fig. 1D,F.

 © The Author(s)

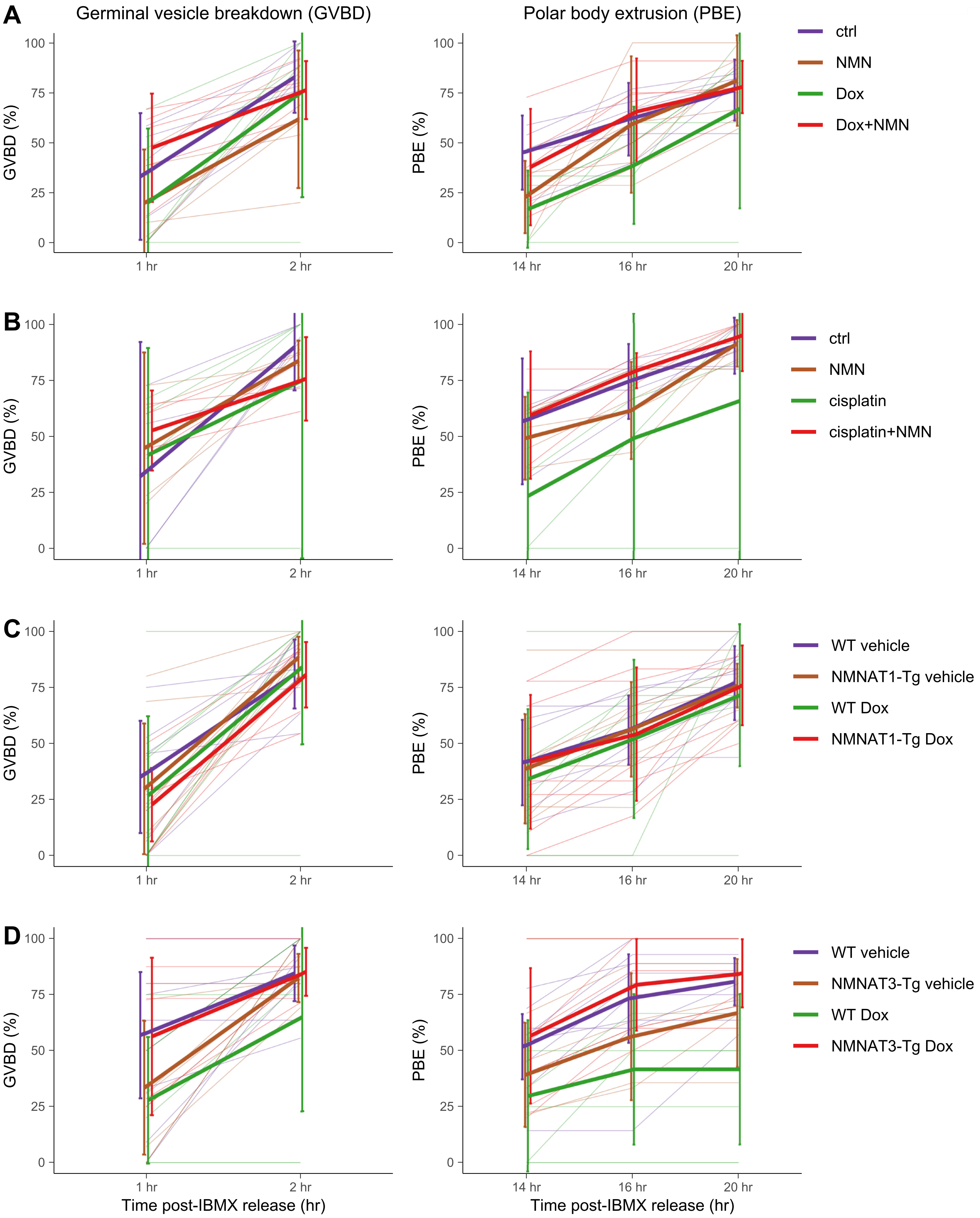
A
Germinal vesicle breakdown (GVBD)
Polar body extrusion (PBE)
ctrl
NMN
Dox
Dox+NMN
B
ctrl
NMN
cisplatin
cisplatin+NMN
C
WT vehicle
NMNAT1-Tg vehicle
WT Dox
NMNAT1-Tg Dox
D
WT vehicle
NMNAT3-Tg vehicle
WT Dox
NMNAT3-Tg Dox
GVBD (%)
PBE (%)
0
25
50
75
100
1 hr
2 hr
14 hr
16 hr
20 hr
Time post-IBMX release (hr)

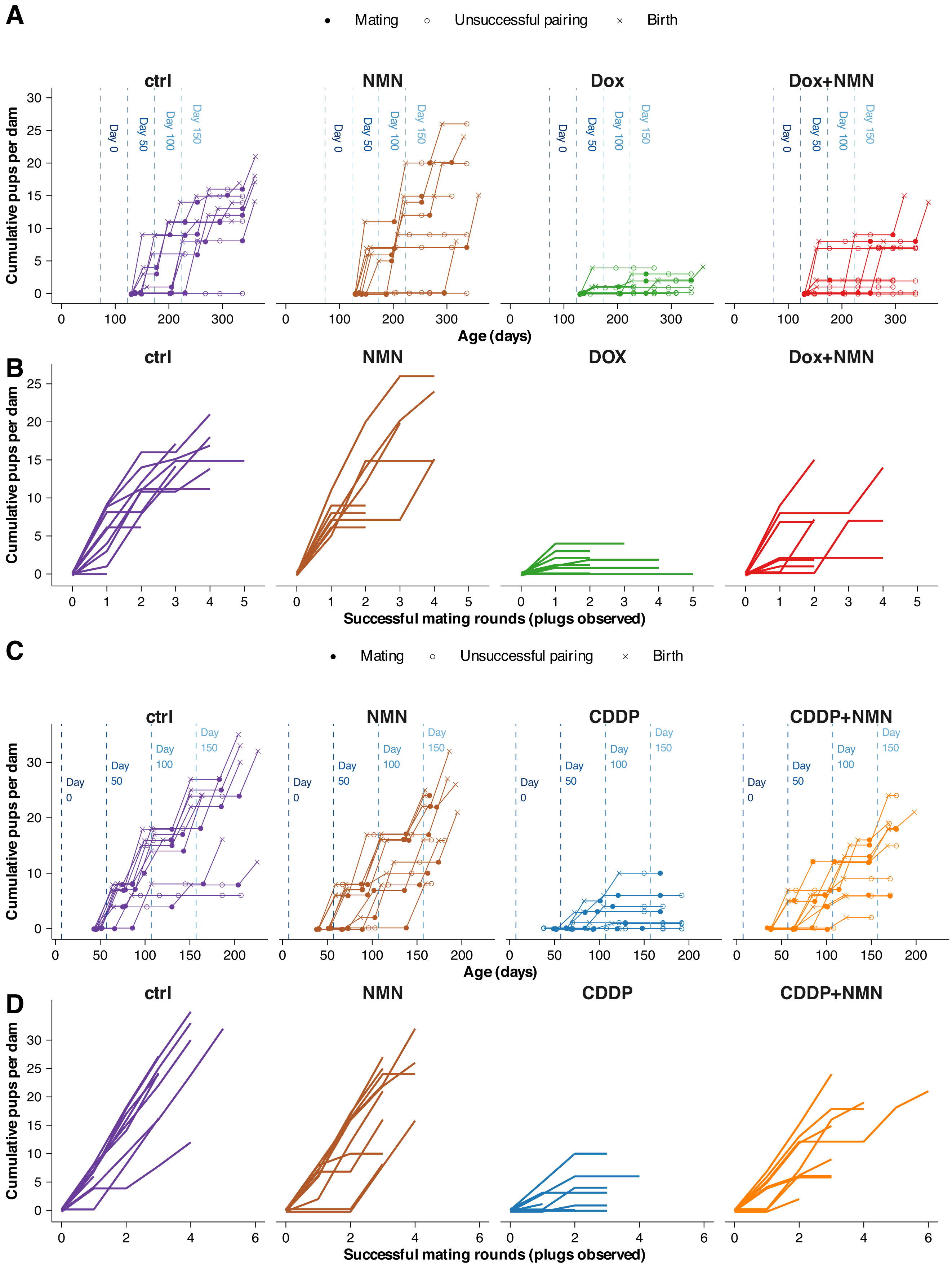
A
Mating
Unsuccessful pairing
Birth
ctrl
NMN
Dox
Dox+NMN
Day 0
Day 50
Day 100
Day 150
Cumulative pups per dam
Age (days)
B
ctrl
NMN
DOX
Dox+NMN
Cumulative pups per dam
Successful mating rounds (plugs observed)
C
Mating
Unsuccessful pairing
Birth
ctrl
NMN
CDDP
CDDP+NMN
Cumulative pups per dam
Age (days)
D
ctrl
NMN
CDDP
CDDP+NMN
Cumulative pups per dam
Successful mating rounds (plugs observed)

 © The Author(s)

Figure EV2. Individualised breeding trial data from Figs. 2 and 3.

(**A**, **B**) Breeding trial data from doxorubicin (Dox) treatment as per Fig. 2A, or (**C**, **D**) from cisplatin (CDDP) treatment as per Fig. 3A. Each line represents the cumulative number of pups from each dam, displayed (**A**, **C**) as a function of time, showing each successful or unsuccessful mating (confirmed by observation of a vaginal plug) and births, as summarised for the entire cohorts in Figs. 2B and 3B. The timing of dox or cisplatin treatment is indicated as Day 0 by a dark blue line, with lighter dashed lines for subsequent 50-day increments. Results are also expressed as (**B**, **D**) pups per dam per round of confirmed mating, as confirmed by the presence of a vaginal plug.

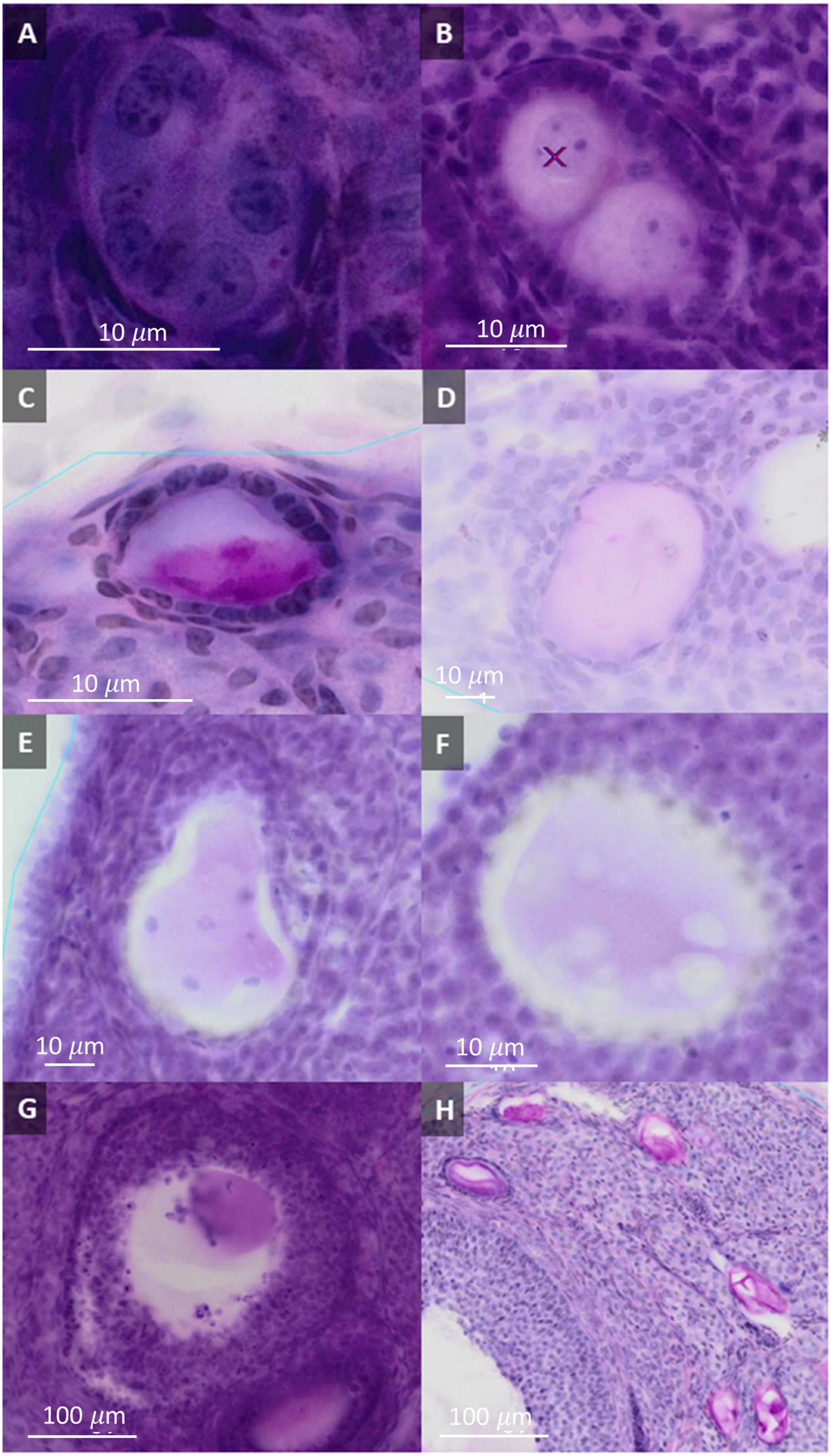

 © The Author(s)

Figure EV3. Representative images of follicles classified as unhealthy during assessments of ovarian reserve (Fig. 3).

Images show follicles with (**A**) no oocyte; (**B**) biovular follicle; (**C**) small follicle with zona pellucida remnants (ZPRs); (**D**) enlarged oocyte with undifferentiated GCs; (**E**) multinuclear oocyte; (**F**) vacuoles present in oocyte; (**G**) atretic follicle; (**H**) ZPRs. Scale bars are as indicated in each image.

© The Author(s)

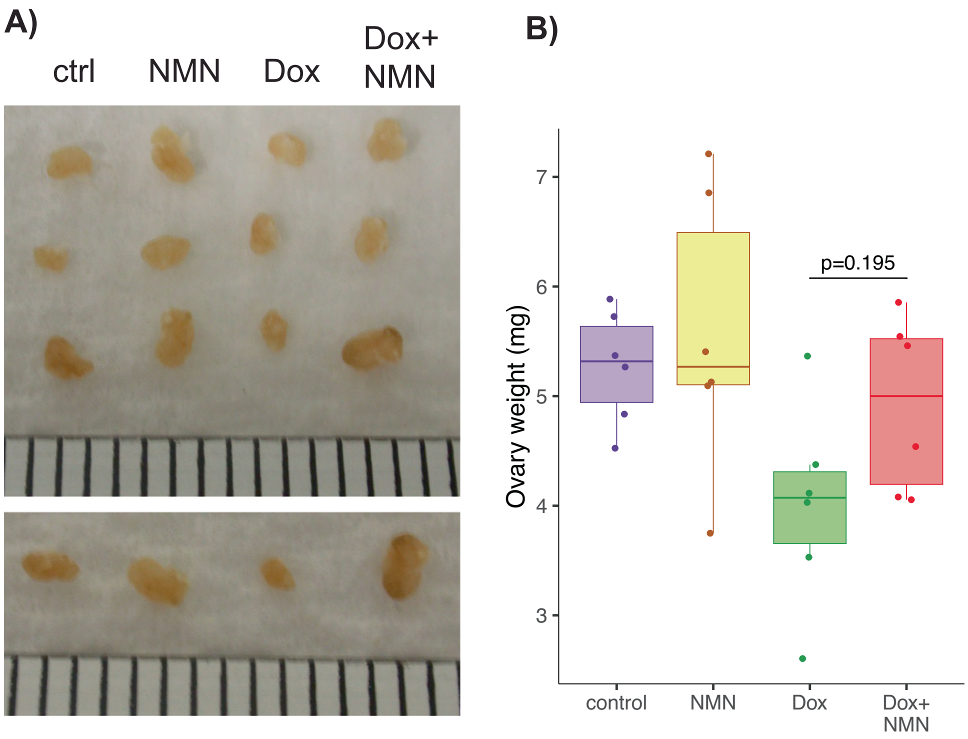

Figure EV4. Whole ovary weight and appearance.

(**A**) Macroscopic appearance of ovaries from non-PMSG stimulated animals treated with doxorubicin (Dox) in the presence or absence of NMN, collected 8 weeks after chemotherapy as described in Figs. 1, 2 and 4. Rulers in pictures show length in millimetres. (**B**) Weights of ovaries collected for stereology from animals treated with doxorubicin (Dox) in the presence or absence of NMN, as described in Fig. 4. $N = 5$–6 animals per treatment. Data were analysed by estimated marginal means for NMN from a linear model of DOX and NMN treatment with Bonferroni correction, *p*-values as indicated.

 © The Author(s)

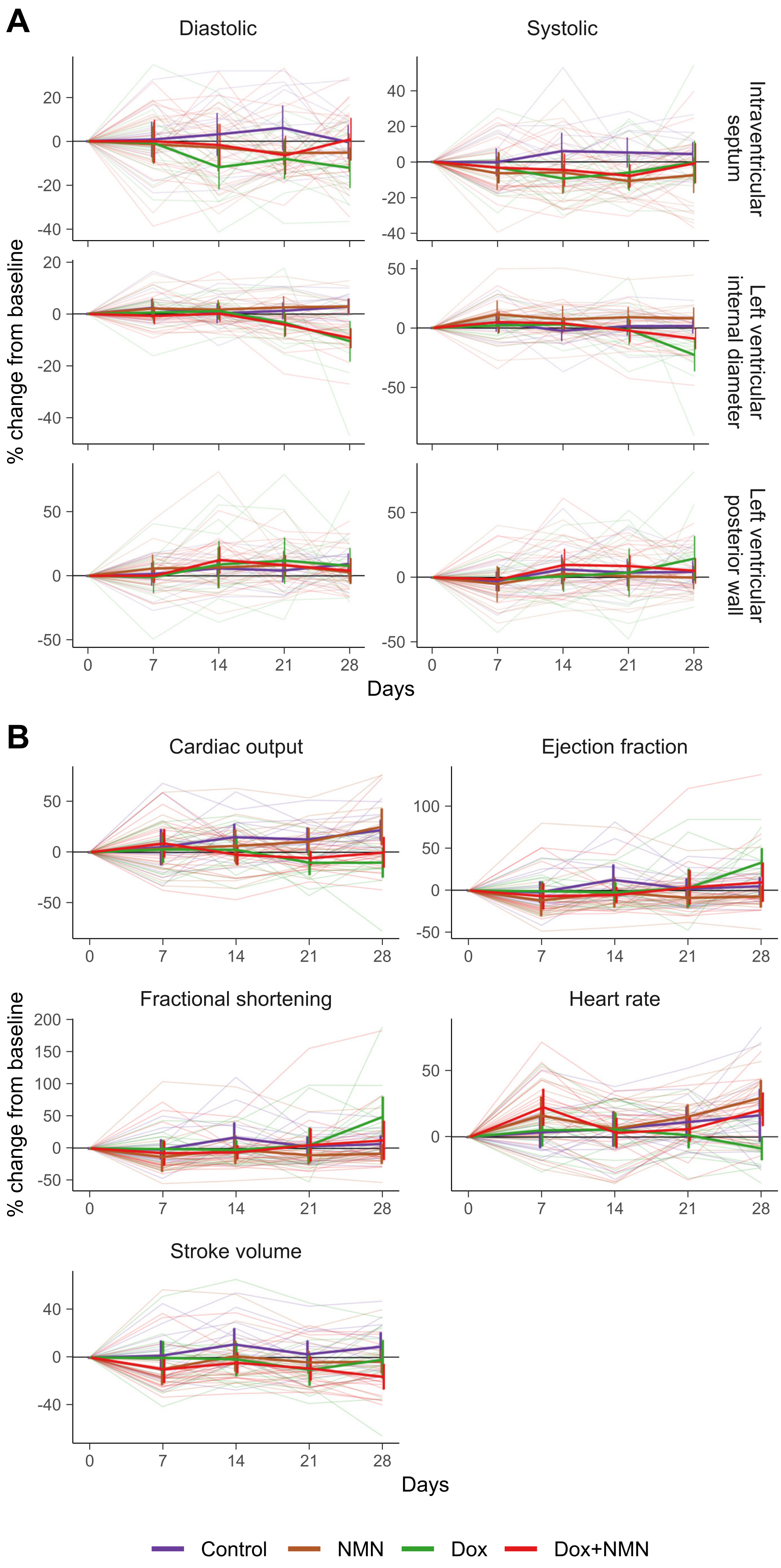
A
Diastolic
Systolic
Intraventricular septum
Left ventricular internal diameter
Left ventricular posterior wall
% change from baseline
Days
B
Cardiac output
Ejection fraction
Fractional shortening
Heart rate
Stroke volume
% change from baseline
Days
Control
NMN
Dox
Dox+NMN

© The Author(s)

Figure EV5. Doxorubicin (DOX) induced cardiotoxicity.

Animals were treated with DOX (8 mg/kg) once per week for 4 weeks, with ultrasound imaging every week to assess parameters of (**A**) left ventricular structure and (**B**) cardiac function. Thick lines indicate the mean values for each treatment group, thin transparent lines indicate data from individual animals. $n = 13$–15 per group, error bars are mean ± 95% CI.

© The Author(s)
